# Thiol-based redox homeostasis and signaling

**DOI:** 10.3389/fpls.2014.00266

**Published:** 2014-06-10

**Authors:** Francisco J. Cejudo, Andreas J. Meyer, Jean-Philippe Reichheld, Nicolas Rouhier, Jose A. Traverso

**Affiliations:** ^1^Instituto de Bioquímica Vegetal y Fotosíntesis, Universidad de Sevilla, Consejo Superior de Investigaciones CientíficasSevilla, Spain; ^2^INRES - Chemical Signalling, University of BonnBonn, Germany; ^3^Laboratoire Génome et Développement des Plantes, Université Perpignan Via DomitiaPerpignan, France; ^4^Laboratoire Génome et Développement des Plantes, CNRSPerpignan, France; ^5^Faculté des Sciences, UMR1136 Université de Lorraine-INRA, Interactions Arbres/Micro-organismesVandoeuvre, France; ^6^Departamento de Biología Celular, Facultad de Ciencias, Universidad de GranadaGranada, Spain; ^7^Estación Experimental del Zaidín, Consejo Superior de Investigaciones CientíficasGranada, Spain

**Keywords:** thioredoxin, glutaredoxin, glutathione, redox regulation, redox signaling, plants

Plants imperatively have to cope with adverse conditions owing to their lack of mobility and to the high amounts of reactive oxygen species (ROS) generated from both respiration and photosynthetic metabolism. Although thiol redox homeostasis in plants is mainly preserved by the cellular glutathione pool, specific strategies have been adopted by the plant kingdom during evolution to manage these “extra” pro-oxidative conditions. Unlike human or yeast, plants generally possess a higher number of genes coding for antioxidant proteins, including protein families responsible of dithiol/disulfide exchange reactions. During the last decades, redox-dependent post-translational modifications of proteins proved to be pivotal to many cellular functions. In particular, this is critically important under some situations of environmental constraints taking into account the alterations and fine adjustment of the cellular redox status occurring during and after any biotic or abiotic stresses.

Indeed, thiol groups of cysteinyl residues are highly sensitive to oxidation which might critically perturb cellular homeostasis. Members of the thioredoxin superfamily are key proteins involved in the regulation of cysteine/protein redox state. They share two common and well-known features: (i) the presence of an active center containing at least one catalytic cysteine residue, and (ii) a highly conserved 3D-structure, the so-called thioredoxin fold, which consists of a four-stranded anti-parallel β-sheet surrounded by three α-helices. Key members of this super family are thioredoxins (TRX) and glutaredoxins (GRX). Representatives of both subgroups are distributed in most cellular compartments and contain at least one TRX motif in their structures. While TRXs are generally reduced by thioredoxin reductases (TR), the reduction of GRXs depends on reduced glutathione (GSH).

The 19 reports of this Research Topic provide timely overviews and new insights into redox regulation, focusing on both TR/TRX and GSH/GRX reduction systems in plants. The biochemical characteristics of these systems as well as their target proteins and functions in metabolic and signaling pathways are discussed. Several contributions to this Research Topic deal with the role of TRX systems in plastid metabolism. Michelet et al. (2013) summarize the seminal contributions reported in the 70s and 80s indicating that several Calvin-Benson cycle enzymes are regulated by the ferredoxin/ferredoxin-thioredoxin-reductase/thioredoxin system. Besides this, based on the observation that multiple redox post-translational modifications including glutathionylation and nitrosylation may affect a single protein, Michelet and collaborators propose that these multiple layers of redox regulation could serve for the fine-tuning of the Calvin-Benson cycle enzyme activities in response to changing conditions. Serrato et al. (2013) reviewed the current knowledge on the functions of plastidial TRXs and discuss their emerging role in non-photosynthetic organs. By analyzing transplastomic tobacco plants overexpressing plastidial TRXs f or m, Rey et al. (2013) show that these types of TRXs fulfill distinct physiological functions and propose a role of TRXm in linking photosynthetic activity, redox homeostasis and antioxidant mechanisms in the chloroplast. In another report, López-Calcagno et al. (2014) discuss the possibility that CP12 proteins are part of a redox-mediated metabolic switch in response to rapid environmental changes, in addition to their classical regulatory role of the Calvin-Benson cycle.

Within the photosynthetic context, Karamoko et al. (2013) propose that the enzymatically assisted sulfhydryl oxidation in plant thylakoid lumen is required for the biogenesis of the energy-transducing membrane systems associated to photosynthesis. Their suggestions are based on both the existence of several luminal disulfide bond containing proteins in *Arabidopsis*, and on the discovery of trans-thylakoid redox pathways controlling disulfide bond formation and reduction.

In addition, the importance of NADPH thioredoxin reductase C (NTRC) in plastid redox regulation is also reported in four articles. The paper by Puerto-Galán et al. (2013) discusses the role of NTRC in the control of the overoxidation status of chloroplast 2-Cys peroxiredoxins (2-Cys-PRX), thus having a crucial function balancing the toxic and signaling activities of hydrogen peroxide. Moreover, Richter and Grimm (2013) emphasize the relevant function of NTRC in conjunction with the FTR/TRX pathway on the redox regulation of tetrapyrrole biosynthesis. This pathway needs to be tightly regulated to adjust chlorophyll content of photosynthetic cells and to avoid the accumulation of chlorophyll biosynthesis intermediates, which may cause oxidative damage. In support of the overarching significance of redox regulation for chloroplast function and adaptation to environmental changes, Chi et al. (2013) discuss the molecular mechanisms and physiological significance of redox-dependent structural changes observed for some proteins including transcriptional factors and co-activators, which switch the chaperone activity of these proteins. Finally, Toivola et al. (2013) report an *in vivo* approach showing the positive effect of NTRC overexpression in plant performance.

The importance of redox regulation in plant mitochondria is also highlighted in this Research Topic by Lázaro et al. (2013). These authors update and discuss the complex antioxidant systems of this organelle, including the complex thiol-based TRX/PRX/Sulfiredoxin (SRX) system. Moreover, these authors focus on the function of this system in the response of plants to abiotic stresses and the regulation by redox post-translational modifications.

Some aspects of the cytosolic redox regulation pathways have also been developed in this Research Topic. Hara and Hisabori (2013) analyzed the kinetics of interactions of a cytosolic TRX *h* with selected target proteins using surface plasmon resonance. The presented data reveal a stronger preference of TRX *h* for an oxidized target, thus explaining the selective association of TRX with oxidized proteins. Zaffagnini et al. (2013) review how cysteine-based modifications of the plant cytosolic glyceraldehyde phosphate dehydrogenase (GAPDH) provoke the inactivation of classical enzymatic activity of this enzyme, but provide additional non-metabolic functions in stress signaling pathways, similar to those that have been identified in animal cells.

GRXs are oxidoreductases of the TRX family which display a particularly rich diversity in higher plants. Beyond classification in three main subgroups based on sequence and structural features, Couturier et al. (2013) indicated that class I GRXs can be subdivided into different groups which can be distinguished by different biochemical and catalytic properties. The importance of a GRX-dependent redox regulation is also evidenced here by Sánchez-Riego et al. (2013) who demonstrate, by performing a physiological and biochemical characterization of single, double and triple *grx* mutants, that GRXs are essential for stress adaptation in cyanobacteria. Finally, Knaff and Sutton (2013) describe an interesting work about the use of GRX to initiate a long-term educational project which will allow examining the structural and biochemical changes in GRXs according to single-point mutational replacements.

Glutathione is a key component in regulation and maintenance of cellular thiol redox homeostasis. It also plays key roles in different aspects of plant development. Here, Rahantaniaina et al. (2013) provide an overview of the main pathways influencing the glutathione redox status and their impact on signaling pathways through regulation of protein thiol status. Schnaubelt et al. (2013) examine the influence of glutathione in plant development and stress tolerance in *Arabidopsis*. The data demonstrate that cellular glutathione homeostasis influences the root architecture and the leaf area under optimal and stress conditions. Another aspect where glutathione and homoglutathione are crucial molecules is nodule development and in the context of legume-rhizobium mutualistic interactions. Indeed, these organs are peculiar due to the formation of a bacteroid in which the oxygen-sensitive nitrogenase reduces di-nitrogen to ammonia. However, the importance of other redox systems in this unique organ has been poorly documented. Frendo et al. (2013) review the current knowledge on the roles played by redox components in nitrogen-fixing symbioses. Finally, Traverso et al. (2013) summarize the insights of the precise involvement of thiol/disulfide-containing proteins at different stages of the sexual plant reproduction, suggesting specific and critical involvement of thiol-based redox modifications in different reproductive processes.

## Conflict of interest statement

The authors declare that the research was conducted in the absence of any commercial or financial relationships that could be construed as a potential conflict of interest.

## References

[B1] Chi Y. H. Paeng S. K. Kim M. J. Hwang G. Y. Melencion S. M. B. Oh H. T. (2013). Redox-dependent functional switching of plant proteins accompanying with their structural changes. Front. Plant Sci. 4:277 10.3389/fpls.2013.00277 23898340 PMC3724125

[B2] Couturier J. Jacquot J. P. Rouhier N. (2013). Toward a refined classification of class I dithiol glutaredoxins from poplar: biochemical basis for the definition of two subclasses. Front. Plant Sci. 4:518 10.3389/fpls.2013.00518 24385978 PMC3866529

[B3] Frendo P. Matamoros M. A. Alloing G. Becana M. (2013). Thiol-based redox signaling in the nitrogen-fixing symbiosis. Front. Plant Sci. 4:376 10.3389/fpls.2013.00376 24133498 PMC3783977

[B4] Hara S. Hisabori T. (2013). Kinetic analysis of the interactions between plant thioredoxin and target proteins. Front. Plant Sci. 4:508 10.3389/fpls.2013.00508 24391652 PMC3867114

[B5] Karamoko M. Gabilly S. T. Hamel P. P. (2013). Operation of trans-thylakoid thiol-metabolizing pathways in photosynthesis. Front. Plant Sci. 4:476 10.3389/fpls.2013.00476 24348486 PMC3842002

[B6] Knaff D. B. Sutton R. B. (2013). Utility of Synechocystis sp. PCC 6803 glutaredoxin A as a platform to study high-resolution mutagenesis of proteins. Front. Plant Sci. 4:461 10.3389/fpls.2013.00461 24298277 PMC3828617

[B7] Lázaro J. J. Jiménez A. Camejo D. Iglesias-Baena I. Martí M. C. Lázaro-Payo A. (2013). Dissecting the integrative antioxidant and redox systems in plant mitochondria. Effect of stress and S-nitrosylation. Front. Plant Sci. 4:460 10.3389/fpls.2013.00460 24348485 PMC3842906

[B8] López-Calcagno P. E. Howard T. P. Raines C. A. (2014). The CP12 protein family: a thioredoxin-mediated metabolic switch?. Front. Plant Sci. 5:9 10.3389/fpls.2014.00009 24523724 PMC3906501

[B9] Michelet L. Zaffagnini M. Morisse S. Sparla F. Pérez-Pérez M. E. Francia F. (2013). Redox regulation of the Calvin–Benson cycle: something old, something new. Front. Plant Sci. 4:470 10.3389/fpls.2013.00470 24324475 PMC3838966

[B10] Puerto-Galán L. Pérez-Ruiz J. M. Ferrández J. Cano B. Naranjo B. Nájera V. A. (2013). Overoxidation of chloroplast 2-Cys peroxiredoxins: balancing toxic and signaling activities of hydrogen peroxide. Front. Plant Sci. 4:310 10.3389/fpls.2013.00310 23967002 PMC3746178

[B11] Rahantaniaina M. S. Tuzet A. Mhamdi A. Noctor G. (2013). Missing links in understanding redox signaling via thiol/disulfide modulation: how is glutathione oxidized in plants? Front. Plant Sci. 4:477 10.3389/fpls.2013.00477 24324478 PMC3838956

[B12] Rey P. Sanz-Barrio R. Innocenti G. Ksas B. Courteille A. Rumeau D. (2013). Overexpression of plastidialthioredoxins f and m differentially alters photosynthetic activity and response to oxidative stress in tobacco plants. Front. Plant Sci. 4:390 10.3389/fpls.2013.00390 24137166 PMC3797462

[B13] Richter A. S. Grimm B. (2013). Thiol-based redox control of enzymes involved in the tetrapyrrole biosynthesis pathway in plants. Front. Plant Sci. 4:371 10.3389/fpls.2013.00371 24065975 PMC3778395

[B14] Sánchez-Riego A. M. López-Maury L. Florencio F. J. (2013). Glutaredoxins are essential for stress adaptation in the cyanobacterium *Synechocystis sp*. PCC 6803. Front. Plant Sci. 4:428 10.3389/fpls.2013.00428 24204369 PMC3816324

[B15] Schnaubelt D. Schulz P. Hannah M. A. Yocgo R. E. Foyer C. H. (2013). A phenomics approach to the analysis of the influence of glutathione on leaf area and abiotic stress tolerance in *Arabidopsis thaliana*. Front. Plant Sci. 4:416 10.3389/fpls.2013.00416 24204368 PMC3817356

[B16] Serrato A. J. Fernández-Trijueque J. Barajas-López J. D. Chueca A. Sahrawy M. (2013). Plastid thioredoxins: a “one-for-all” redox-signaling system in plants. Front. Plant Sci. 4:463 10.3389/fpls.2013.00463 24319449 PMC3836485

[B17] Toivola J. Nikkanen L. Dahlström K. M. Salminen T. A. Lepistö A. Vignols F. (2013). Overexpression of chloroplast NADPH-dependent thioredoxin reductase in Arabidopsis enhances leaf growth and elucidates *in vivo* function of reductase and thioredoxin domains. Front. Plant Sci. 4:389 10.3389/fpls.2013.00389 24115951 PMC3792407

[B18] Traverso J. A. Pulido A. Rodríguez-García M. I. Alché J. D. (2013). Thiol-based redox regulation in sexual plant reproduction: new insights and perspectives. Front. Plant Sci. 4:465 10.3389/fpls.2013.00465 24294217 PMC3827552

[B19] Zaffagnini M. Fermani S. Costa A. Lemaire S. D. Trost P. (2013). Plant cytoplasmic GAPDH: redox post-translational modifications and moonlighting properties. Front. Plant Sci. 4:450 10.3389/fpls.2013.00450 24282406 PMC3824636

